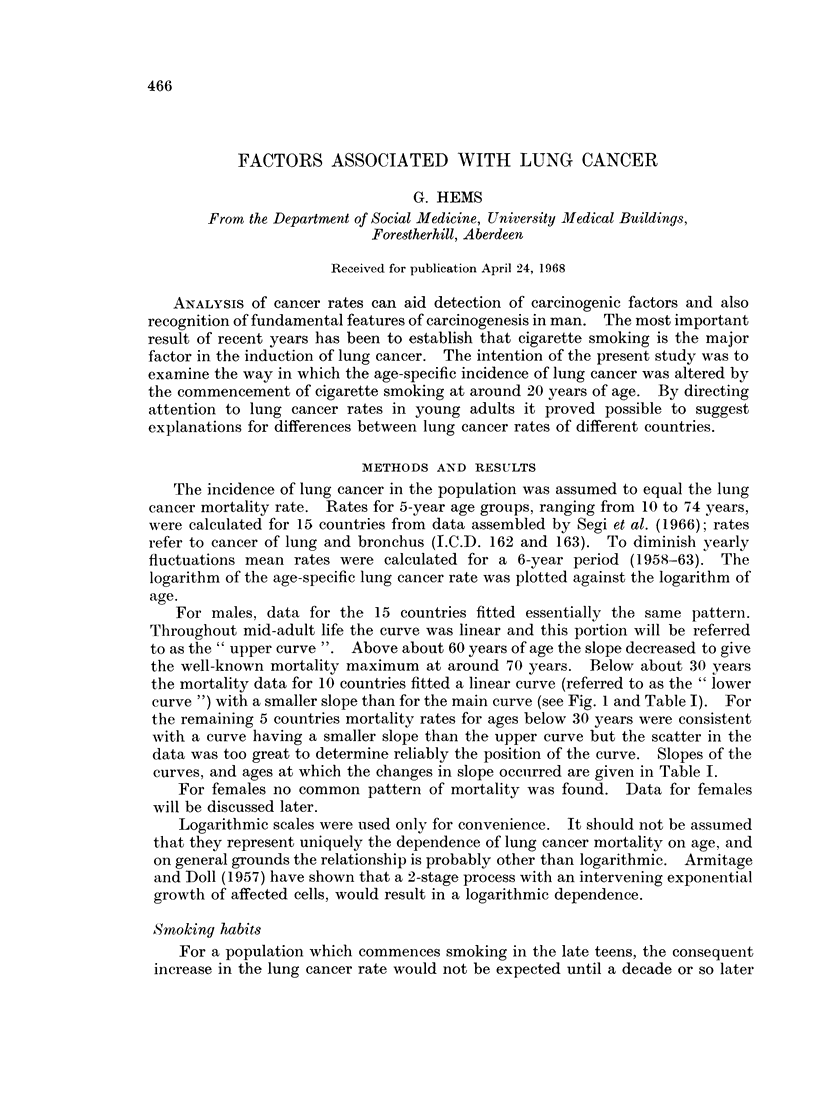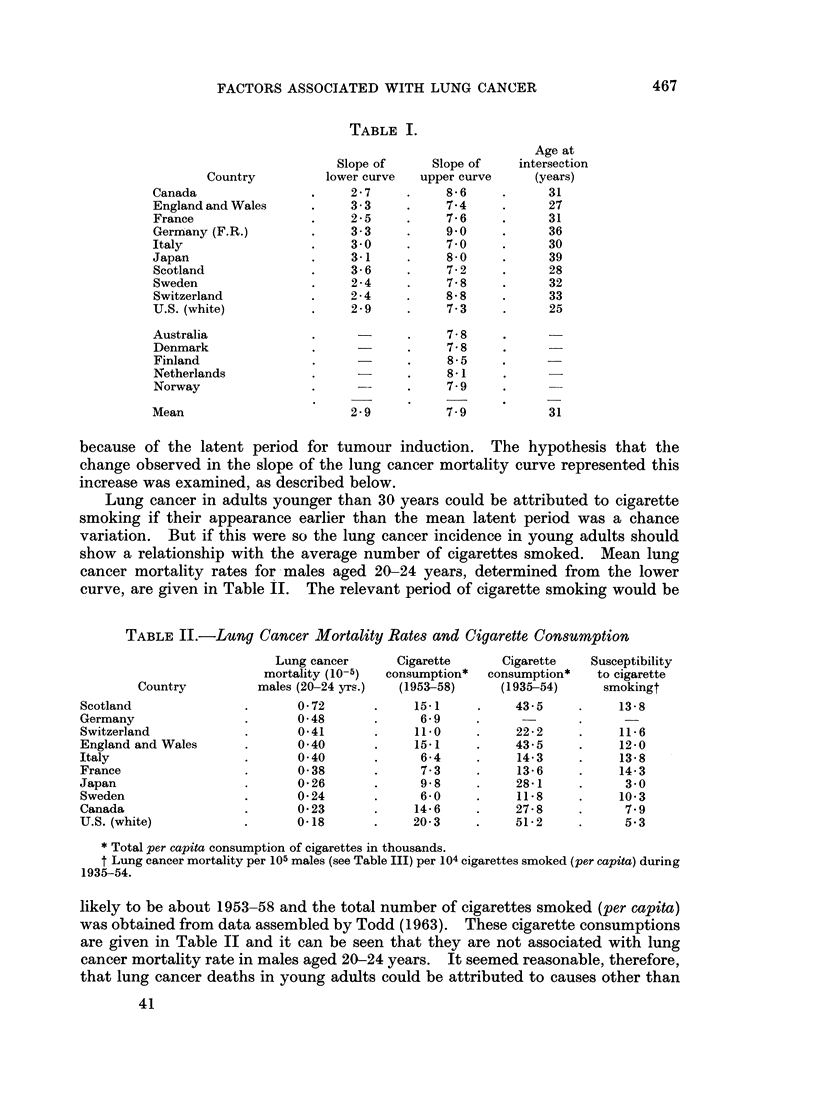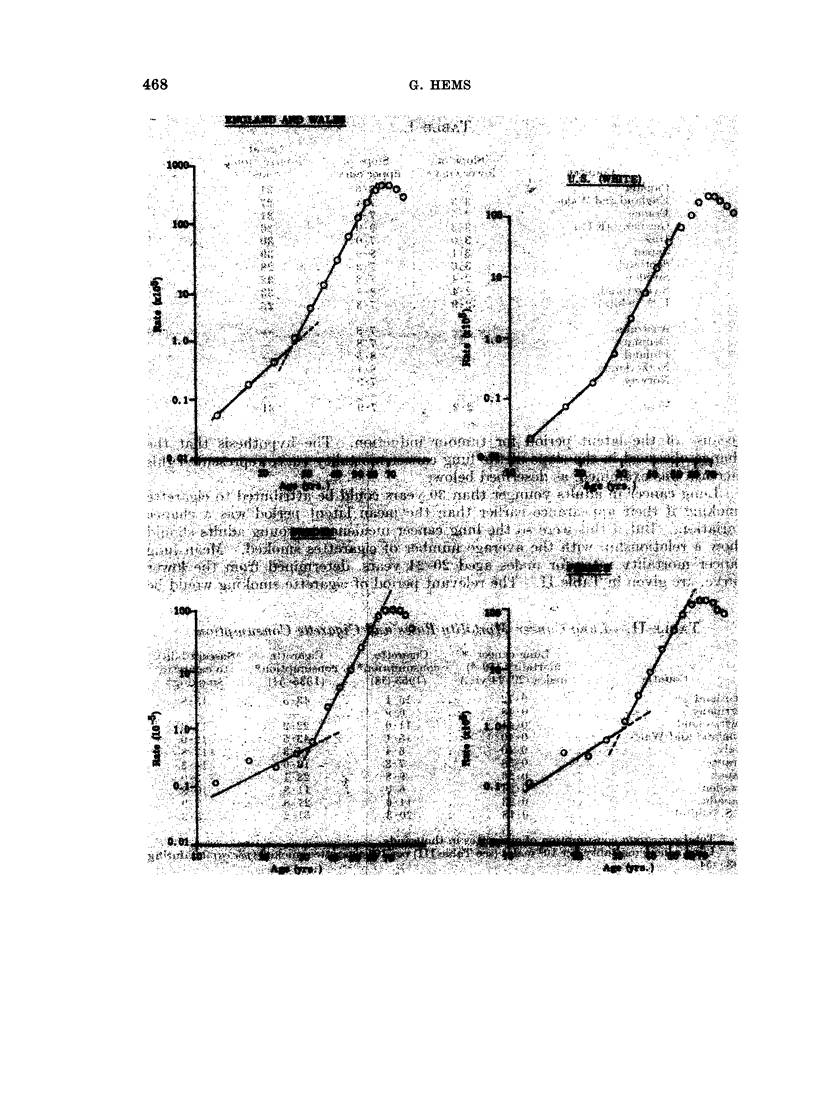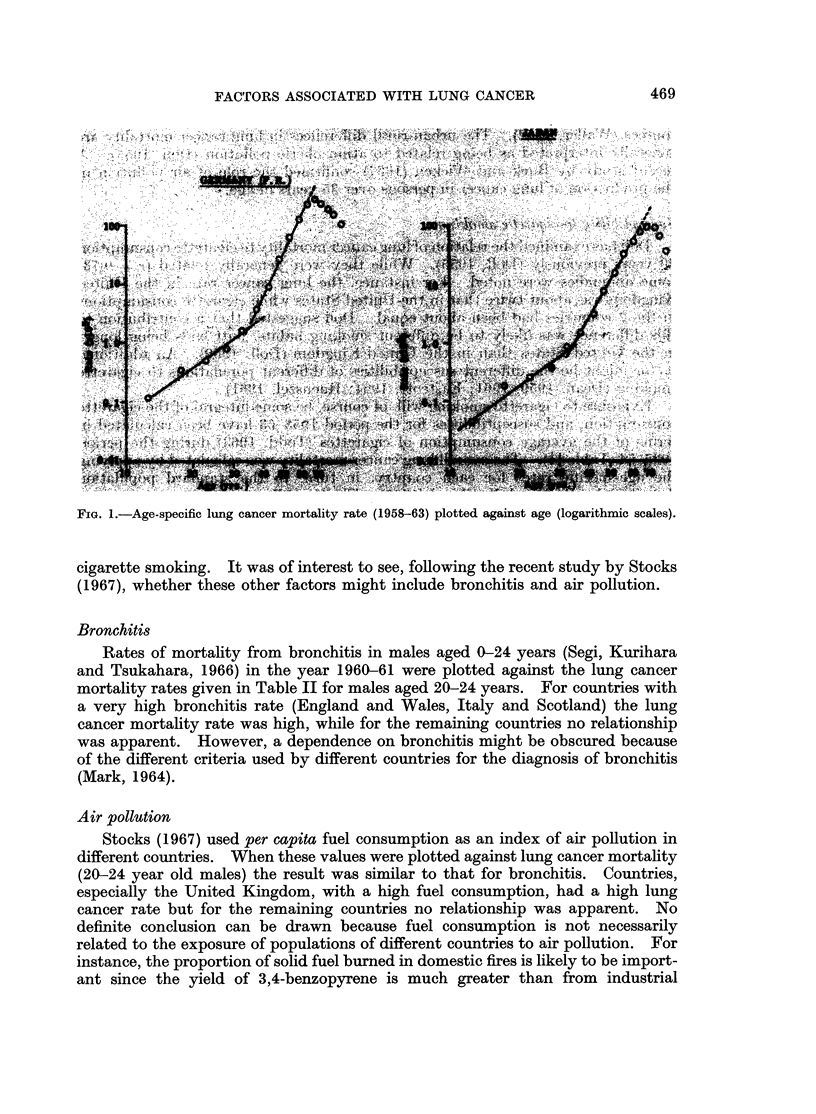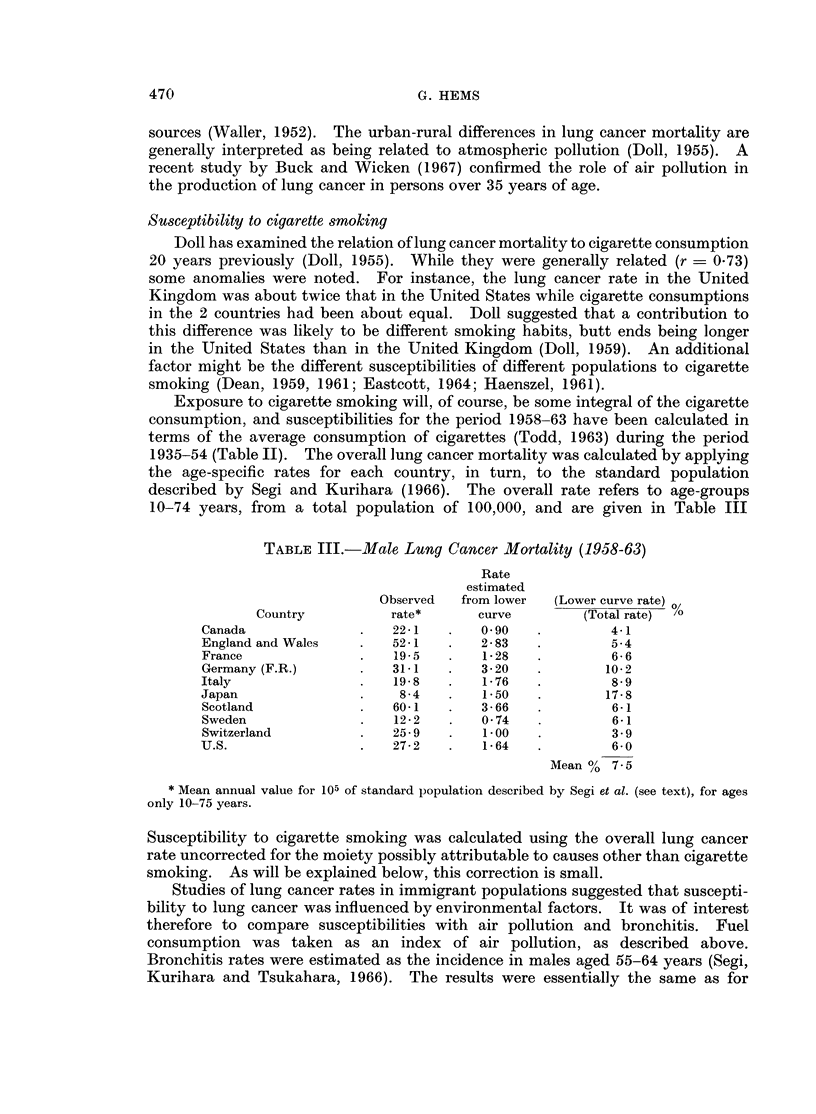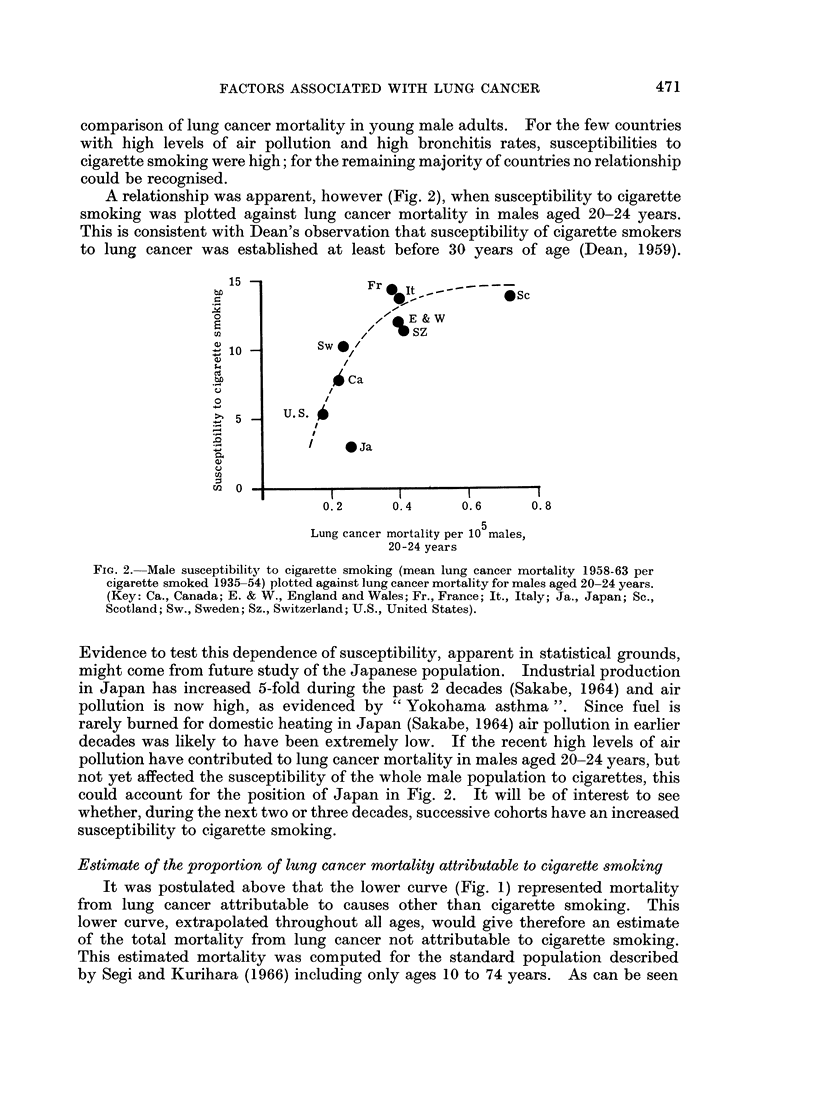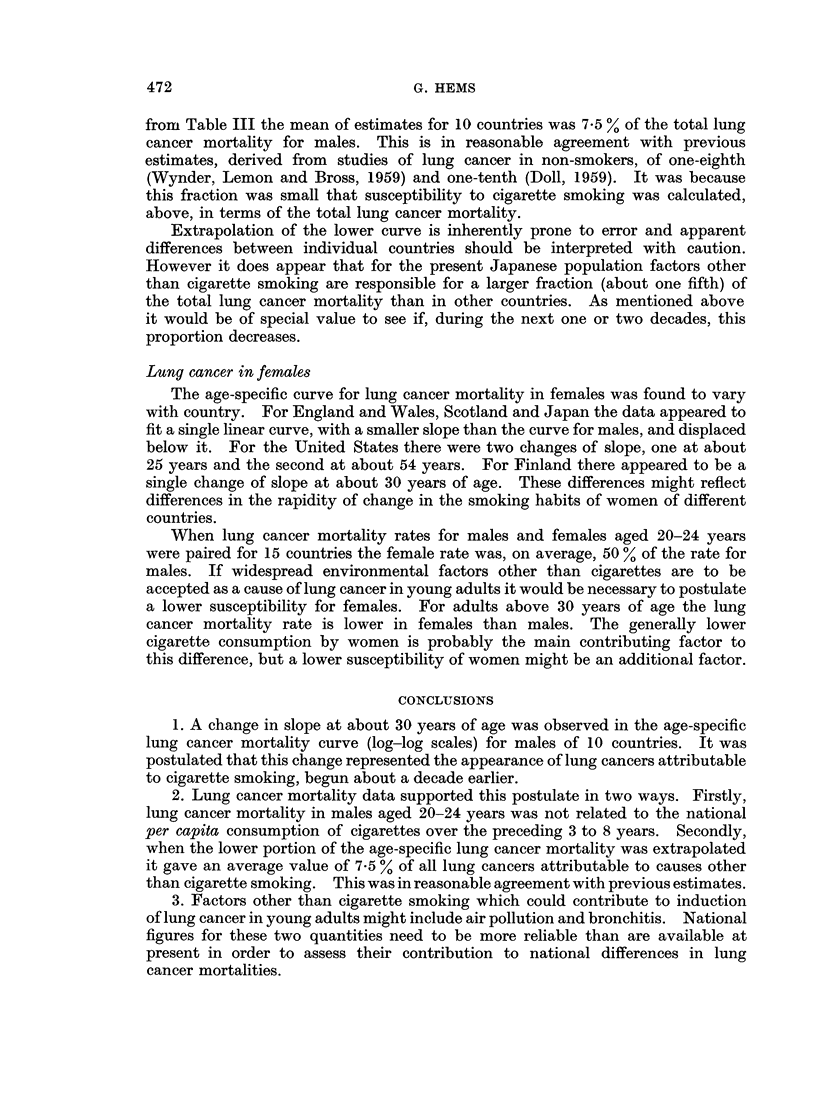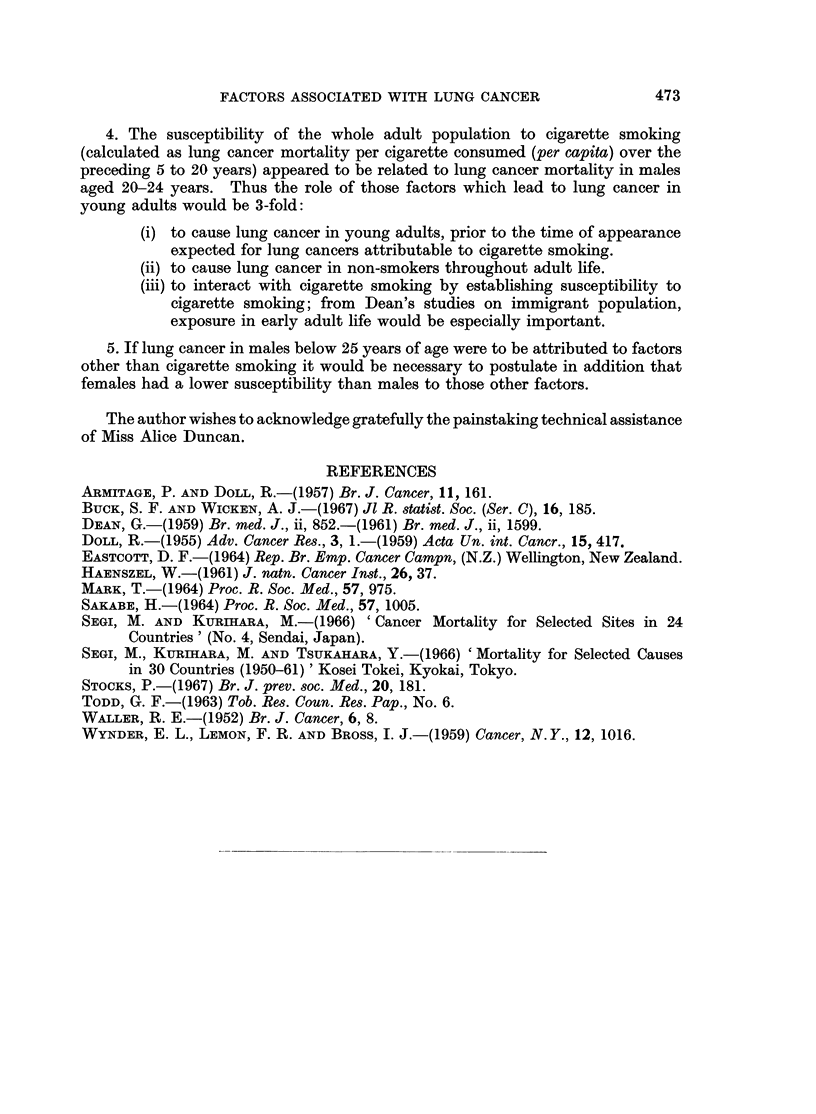# Factors associated with lung cancer.

**DOI:** 10.1038/bjc.1968.54

**Published:** 1968-09

**Authors:** G. Hems


### FACTORS ASSOCIATED WITH LUNG CANCER

G. HEMS

*From the Department of Social Medicine, University Medical Buildings, Forestherhill, Aberdeen*

Received for publication April 24, 1968

ANALYSIS of cancer rates can aid detection of carcinogenic factors and also recognition of fundamental features of carcinogenesis in man. The most important result of recent years has been to establish that cigarette smoking is the major factor in the induction of lung cancer. The intention of the present study was to examine the way in which the age-specific incidence of lung cancer was altered by the commencement of cigarette smoking at around 20 years of age. By directing attention to lung cancer rates in young adults it proved possible to suggest explanations for differences between lung cancer rates of different countries.

### METHODS AND RESULTS

The incidence of lung cancer in the population was assumed to equal the lung cancer mortality rate. Rates for 5-year age groups, ranging from 10 to 74 years, were calculated for 15 countries from data assembled by Segi *et al.* (1966); rates refer to cancer of lung and bronchus (I.C.D. 162 and 163). To diminish yearly fluctuations mean rates were calculated for a 6-year period (1958–63). The logarithm of the age-specific lung cancer rate was plotted against the logarithm of age.

For males, data for the 15 countries fitted essentially the same pattern. Throughout mid-adult life the curve was linear and this portion will be referred to as the " upper curve ". Above about 60 years of age the slope decreased to give the well-known mortality maximum at around 70 years. Below about 30 years the mortality data for 10 countries fitted a linear curve (referred to as the " lower curve ") with a smaller slope than for the main curve (see Fig. 1 and Table I). For the remaining 5 countries mortality rates for ages below 30 years were consistent with a curve having a smaller slope than the upper curve but the scatter in the data was too great to determine reliably the position of the curve. Slopes of the curves, and ages at which the changes in slope occurred are given in Table I.

For females no common pattern of mortality was found. Data for females will be discussed later.

Logarithmic scales were used only for convenience. It should not be assumed that they represent uniquely the dependence of lung cancer mortality on age, and on general grounds the relationship is probably other than logarithmic. Armitage and Doll (1957) have shown that a 2-stage process with an intervening exponential growth of affected cells, would result in a logarithmic dependence.

### *Smoking habits*

For a population which commences smoking in the late teens, the consequent increase in the lung cancer rate would not be expected until a decade or so later

TABLE I.

| Country | Slope of lower curve | Slope of upper curve | Age at intersection (years) |
|---|---|---|---|
| Canada | 2·7 | 8·6 | 31 |
| England and Wales | 3·3 | 7·4 | 27 |
| France | 2·5 | 7·6 | 31 |
| Germany (F.R.) | 3·3 | 9·0 | 36 |
| Italy | 3·0 | 7·0 | 30 |
| Japan | 3·1 | 8·0 | 39 |
| Scotland | 3·6 | 7·2 | 28 |
| Sweden | 2·4 | 7·8 | 32 |
| Switzerland | 2·4 | 8·8 | 33 |
| U.S. (white) | 2·9 | 7·3 | 25 |
| Australia | — | 7·8 | — |
| Denmark | — | 7·8 | — |
| Finland | — | 8·5 | — |
| Netherlands | — | 8·1 | — |
| Norway | — | 7·9 | — |
| Mean | 2·9 | 7·9 | 31 |

because of the latent period for tumour induction. The hypothesis that the change observed in the slope of the lung cancer mortality curve represented this increase was examined, as described below.

Lung cancer in adults younger than 30 years could be attributed to cigarette smoking if their appearance earlier than the mean latent period was a chance variation. But if this were so the lung cancer incidence in young adults should show a relationship with the average number of cigarettes smoked. Mean lung cancer mortality rates for males aged 20–24 years, determined from the lower curve, are given in Table II. The relevant period of cigarette smoking would be

TABLE II.—*Lung Cancer Mortality Rates and Cigarette Consumption*

| Country | Lung cancer mortality ($10^{-5}$) males (20–24 yrs.) | Cigarette consumption* (1953–58) | Cigarette consumption* (1935–54) | Susceptibility to cigarette smoking† |
|---|---|---|---|---|
| Scotland | 0·72 | 15·1 | 43·5 | 13·8 |
| Germany | 0·48 | 6·9 | — | — |
| Switzerland | 0·41 | 11·0 | 22·2 | 11·6 |
| England and Wales | 0·40 | 15·1 | 43·5 | 12·0 |
| Italy | 0·40 | 6·4 | 14·3 | 13·8 |
| France | 0·38 | 7·3 | 13·6 | 14·3 |
| Japan | 0·26 | 9·8 | 28·1 | 3·0 |
| Sweden | 0·24 | 6·0 | 11·8 | 10·3 |
| Canada | 0·23 | 14·6 | 27·8 | 7·9 |
| U.S. (white) | 0·18 | 20·3 | 51·2 | 5·3 |

* Total *per capita* consumption of cigarettes in thousands.
† Lung cancer mortality per $10^5$ males (see Table III) per $10^4$ cigarettes smoked (*per capita*) during 1935–54.

likely to be about 1953–58 and the total number of cigarettes smoked (*per capita*) was obtained from data assembled by Todd (1963). These cigarette consumptions are given in Table II and it can be seen that they are not associated with lung cancer mortality rate in males aged 20–24 years. It seemed reasonable, therefore, that lung cancer deaths in young adults could be attributed to causes other than

ENGLAND AND WALES

U.S. (WHITE)

SWEDEN

FRANCE

Rate (x10$^5$)

Rate (10$^{-5}$)

1000

100

10

1.0

0.1

0.01

10

20

30

40

50 60 70

Age (yrs.)

FIG. 1.—Age-specific lung cancer mortality rate (1958–63) plotted against age (logarithmic scales).

cigarette smoking. It was of interest to see, following the recent study by Stocks (1967), whether these other factors might include bronchitis and air pollution.

*Bronchitis*

Rates of mortality from bronchitis in males aged 0–24 years (Segi, Kurihara and Tsukahara, 1966) in the year 1960–61 were plotted against the lung cancer mortality rates given in Table II for males aged 20–24 years. For countries with a very high bronchitis rate (England and Wales, Italy and Scotland) the lung cancer mortality rate was high, while for the remaining countries no relationship was apparent. However, a dependence on bronchitis might be obscured because of the different criteria used by different countries for the diagnosis of bronchitis (Mark, 1964).

*Air pollution*

Stocks (1967) used *per capita* fuel consumption as an index of air pollution in different countries. When these values were plotted against lung cancer mortality (20–24 year old males) the result was similar to that for bronchitis. Countries, especially the United Kingdom, with a high fuel consumption, had a high lung cancer rate but for the remaining countries no relationship was apparent. No definite conclusion can be drawn because fuel consumption is not necessarily related to the exposure of populations of different countries to air pollution. For instance, the proportion of solid fuel burned in domestic fires is likely to be important since the yield of 3,4-benzopyrene is much greater than from industrial sources (Waller, 1952). The urban-rural differences in lung cancer mortality are generally interpreted as being related to atmospheric pollution (Doll, 1955). A recent study by Buck and Wicken (1967) confirmed the role of air pollution in the production of lung cancer in persons over 35 years of age.

*Susceptibility to cigarette smoking*

Doll has examined the relation of lung cancer mortality to cigarette consumption 20 years previously (Doll, 1955). While they were generally related ($r = 0{\cdot}73$) some anomalies were noted. For instance, the lung cancer rate in the United Kingdom was about twice that in the United States while cigarette consumptions in the 2 countries had been about equal. Doll suggested that a contribution to this difference was likely to be different smoking habits, butt ends being longer in the United States than in the United Kingdom (Doll, 1959). An additional factor might be the different susceptibilities of different populations to cigarette smoking (Dean, 1959, 1961; Eastcott, 1964; Haenszel, 1961).

Exposure to cigarette smoking will, of course, be some integral of the cigarette consumption, and susceptibilities for the period 1958–63 have been calculated in terms of the average consumption of cigarettes (Todd, 1963) during the period 1935–54 (Table II). The overall lung cancer mortality was calculated by applying the age-specific rates for each country, in turn, to the standard population described by Segi and Kurihara (1966). The overall rate refers to age-groups 10–74 years, from a total population of 100,000, and are given in Table III

TABLE III.—*Male Lung Cancer Mortality (1958-63)*

| Country | Observed rate* | Rate estimated from lower curve | (Lower curve rate)/(Total rate) % |
|---|---|---|---|
| Canada | 22·1 | 0·90 | 4·1 |
| England and Wales | 52·1 | 2·83 | 5·4 |
| France | 19·5 | 1·28 | 6·6 |
| Germany (F.R.) | 31·1 | 3·20 | 10·2 |
| Italy | 19·8 | 1·76 | 8·9 |
| Japan | 8·4 | 1·50 | 17·8 |
| Scotland | 60·1 | 3·66 | 6·1 |
| Sweden | 12·2 | 0·74 | 6·1 |
| Switzerland | 25·9 | 1·00 | 3·9 |
| U.S. | 27·2 | 1·64 | 6·0 |
| | | Mean % | 7·5 |

* Mean annual value for $10^5$ of standard population described by Segi *et al.* (see text), for ages only 10–75 years.

Susceptibility to cigarette smoking was calculated using the overall lung cancer rate uncorrected for the moiety possibly attributable to causes other than cigarette smoking. As will be explained below, this correction is small.

Studies of lung cancer rates in immigrant populations suggested that susceptibility to lung cancer was influenced by environmental factors. It was of interest therefore to compare susceptibilities with air pollution and bronchitis. Fuel consumption was taken as an index of air pollution, as described above. Bronchitis rates were estimated as the incidence in males aged 55–64 years (Segi, Kurihara and Tsukahara, 1966). The results were essentially the same as for comparison of lung cancer mortality in young male adults. For the few countries with high levels of air pollution and high bronchitis rates, susceptibilities to cigarette smoking were high; for the remaining majority of countries no relationship could be recognised.

A relationship was apparent, however (Fig. 2), when susceptibility to cigarette smoking was plotted against lung cancer mortality in males aged 20–24 years. This is consistent with Dean's observation that susceptibility of cigarette smokers to lung cancer was established at least before 30 years of age (Dean, 1959).

FIG. 2.—Male susceptibility to cigarette smoking (mean lung cancer mortality 1958-63 per cigarette smoked 1935–54) plotted against lung cancer mortality for males aged 20–24 years. (Key: Ca., Canada; E. & W., England and Wales; Fr., France; It., Italy; Ja., Japan; Sc., Scotland; Sw., Sweden; Sz., Switzerland; U.S., United States).

Evidence to test this dependence of susceptibility, apparent in statistical grounds, might come from future study of the Japanese population. Industrial production in Japan has increased 5-fold during the past 2 decades (Sakabe, 1964) and air pollution is now high, as evidenced by " Yokohama asthma ". Since fuel is rarely burned for domestic heating in Japan (Sakabe, 1964) air pollution in earlier decades was likely to have been extremely low. If the recent high levels of air pollution have contributed to lung cancer mortality in males aged 20–24 years, but not yet affected the susceptibility of the whole male population to cigarettes, this could account for the position of Japan in Fig. 2. It will be of interest to see whether, during the next two or three decades, successive cohorts have an increased susceptibility to cigarette smoking.

*Estimate of the proportion of lung cancer mortality attributable to cigarette smoking*

It was postulated above that the lower curve (Fig. 1) represented mortality from lung cancer attributable to causes other than cigarette smoking. This lower curve, extrapolated throughout all ages, would give therefore an estimate of the total mortality from lung cancer not attributable to cigarette smoking. This estimated mortality was computed for the standard population described by Segi and Kurihara (1966) including only ages 10 to 74 years. As can be seen from Table III the mean of estimates for 10 countries was 7·5 % of the total lung cancer mortality for males. This is in reasonable agreement with previous estimates, derived from studies of lung cancer in non-smokers, of one-eighth (Wynder, Lemon and Bross, 1959) and one-tenth (Doll, 1959). It was because this fraction was small that susceptibility to cigarette smoking was calculated, above, in terms of the total lung cancer mortality.

Extrapolation of the lower curve is inherently prone to error and apparent differences between individual countries should be interpreted with caution. However it does appear that for the present Japanese population factors other than cigarette smoking are responsible for a larger fraction (about one fifth) of the total lung cancer mortality than in other countries. As mentioned above it would be of special value to see if, during the next one or two decades, this proportion decreases.

*Lung cancer in females*

The age-specific curve for lung cancer mortality in females was found to vary with country. For England and Wales, Scotland and Japan the data appeared to fit a single linear curve, with a smaller slope than the curve for males, and displaced below it. For the United States there were two changes of slope, one at about 25 years and the second at about 54 years. For Finland there appeared to be a single change of slope at about 30 years of age. These differences might reflect differences in the rapidity of change in the smoking habits of women of different countries.

When lung cancer mortality rates for males and females aged 20–24 years were paired for 15 countries the female rate was, on average, 50 % of the rate for males. If widespread environmental factors other than cigarettes are to be accepted as a cause of lung cancer in young adults it would be necessary to postulate a lower susceptibility for females. For adults above 30 years of age the lung cancer mortality rate is lower in females than males. The generally lower cigarette consumption by women is probably the main contributing factor to this difference, but a lower susceptibility of women might be an additional factor.

### CONCLUSIONS

1. A change in slope at about 30 years of age was observed in the age-specific lung cancer mortality curve (log–log scales) for males of 10 countries. It was postulated that this change represented the appearance of lung cancers attributable to cigarette smoking, begun about a decade earlier.

2. Lung cancer mortality data supported this postulate in two ways. Firstly, lung cancer mortality in males aged 20–24 years was not related to the national *per capita* consumption of cigarettes over the preceding 3 to 8 years. Secondly, when the lower portion of the age-specific lung cancer mortality was extrapolated it gave an average value of 7·5 % of all lung cancers attributable to causes other than cigarette smoking. This was in reasonable agreement with previous estimates.

3. Factors other than cigarette smoking which could contribute to induction of lung cancer in young adults might include air pollution and bronchitis. National figures for these two quantities need to be more reliable than are available at present in order to assess their contribution to national differences in lung cancer mortalities.

4. The susceptibility of the whole adult population to cigarette smoking (calculated as lung cancer mortality per cigarette consumed (*per capita*) over the preceding 5 to 20 years) appeared to be related to lung cancer mortality in males aged 20–24 years. Thus the role of those factors which lead to lung cancer in young adults would be 3-fold:

(i) to cause lung cancer in young adults, prior to the time of appearance expected for lung cancers attributable to cigarette smoking.
(ii) to cause lung cancer in non-smokers throughout adult life.
(iii) to interact with cigarette smoking by establishing susceptibility to cigarette smoking; from Dean's studies on immigrant population, exposure in early adult life would be especially important.

5. If lung cancer in males below 25 years of age were to be attributed to factors other than cigarette smoking it would be necessary to postulate in addition that females had a lower susceptibility than males to those other factors.

The author wishes to acknowledge gratefully the painstaking technical assistance of Miss Alice Duncan.

### REFERENCES

Armitage, P. and Doll, R.—(1957) *Br. J. Cancer*, **11**, 161.
Buck, S. F. and Wicken, A. J.—(1967) *Jl R. statist. Soc.* (*Ser. C*), **16**, 185.
Dean, G.—(1959) *Br. med. J.*, ii, 852.—(1961) *Br. med. J.*, ii, 1599.
Doll, R.—(1955) *Adv. Cancer Res.*, **3**, 1.—(1959) *Acta Un. int. Cancr.*, **15**, 417.
Eastcott, D. F.—(1964) *Rep. Br. Emp. Cancer Campn*, (N.Z.) Wellington, New Zealand.
Haenszel, W.—(1961) *J. natn. Cancer Inst.*, **26**, 37.
Mark, T.—(1964) *Proc. R. Soc. Med.*, **57**, 975.
Sakabe, H.—(1964) *Proc. R. Soc. Med.*, **57**, 1005.
Segi, M. and Kurihara, M.—(1966) 'Cancer Mortality for Selected Sites in 24 Countries' (No. 4, Sendai, Japan).
Segi, M., Kurihara, M. and Tsukahara, Y.—(1966) 'Mortality for Selected Causes in 30 Countries (1950–61)' Kosei Tokei, Kyokai, Tokyo.
Stocks, P.—(1967) *Br. J. prev. soc. Med.*, **20**, 181.
Todd, G. F.—(1963) *Tob. Res. Coun. Res. Pap.*, No. 6.
Waller, R. E.—(1952) *Br. J. Cancer*, **6**, 8.
Wynder, E. L., Lemon, F. R. and Bross, I. J.—(1959) *Cancer, N.Y.*, **12**, 1016.